# A Heparan-Dependent Herpesvirus Targets the Olfactory Neuroepithelium for Host Entry

**DOI:** 10.1371/journal.ppat.1002986

**Published:** 2012-11-01

**Authors:** Ricardo Milho, Bruno Frederico, Stacey Efstathiou, Philip G. Stevenson

**Affiliations:** Division of Virology, Department of Pathology, University of Cambridge, Cambridge, United Kingdom; University of California, Berkeley, United States of America

## Abstract

Herpesviruses are ubiquitous pathogens that cause much disease. The difficulty of clearing their established infections makes host entry an important target for control. However, while herpesviruses have been studied extensively *in vitro*, how they cross differentiated mucus-covered epithelia *in vivo* is unclear. To establish general principles we tracked host entry by Murid Herpesvirus-4 (MuHV-4), a lymphotropic rhadinovirus related to the Kaposi's Sarcoma-associated Herpesvirus. Spontaneously acquired virions targeted the olfactory neuroepithelium. Like many herpesviruses, MuHV-4 binds to heparan sulfate (HS), and virions unable to bind HS showed poor host entry. While the respiratory epithelium expressed only basolateral HS and was bound poorly by incoming virions, the neuroepithelium also displayed HS on its apical neuronal cilia and was bound strongly. Incoming virions tracked down the neuronal cilia, and either infected neurons or reached the underlying microvilli of the adjacent glial (sustentacular) cells and infected them. Thus the olfactory neuroepithelium provides an important and complex site of HS-dependent herpesvirus uptake.

## Author Summary

Herpesviruses are supremely successful mammalian parasites. Yet their infections rarely present until well established, so how new hosts are first infected has been unclear. Understanding this is likely to be crucial for infection control. Using Murid Herpesvirus-4, a relative of the Kaposi's Sarcoma-associated Herpesvirus, we identified the olfactory neuroepithelium as a major portal of host entry. Heparan sulfate (HS) binding, which is common to many herpesviruses, played a key role. The HS of most epithelia is solely basolateral and therefore inaccessible to incoming, apical virions. The neuroepithelium, by contrast, also displayed HS on its apical surface. This comprises a dense meshwork of the neuronal cilia that mediate olfaction. Incoming virions bound to the cilia, as did a recombinant form of the virion glycoprotein H/L heterodimer. Some virions tracked down the cilia to infect neurons. Others were transferred to the microvilli of adjacent sustentacular cells. The central role of HS in this first detailed description of host entry by a mammalian herpesvirus, and the paucity of accessible HS on other epithelia, suggested that many HS-binding herpesviruses could follow a similar path.

## Introduction

The difficulty of clearing latent herpesvirus infections makes host entry a key target for disease control. However, how herpesviruses first infect new hosts remains ill-defined. Salivary virus shedding is common [1], and the oral symptoms of some primary human herpesvirus infections have been interpreted as host entry being oral [2]. However clinical presentation occurs relatively late in infection - for example glandular fever post-dates Epstein-Barr virus (EBV) host entry by at least a month [3]. Thus it is more likely to reflect host exit than entry. Herpesvirus latency reservoirs generally incorporate a capacity for viral dissemination, so the routes used for entry and exit are not necessarily the same; for example the cutaneous blisters of acute Varicella-Zoster virus infection are a dedicated exit route. Clinical presentations therefore have only a limited capacity to reveal how infection first occurs.

The need to define host entry functionally rather than descriptively makes experimentally accessible viruses such as Murid Herpesvirus-4 (MuHV-4) [4]–[7] important sources of information. This natural parasite of yellow-necked mice (*Apodemus flavicollis*) [8] is closely related to the Kaposi's Sarcoma-associated Herpesvirus (KSHV) [9], [10]. MuHV-4, KSHV and EBV all persist in B cells [11]. When MuHV-4 is delivered intranasally to anesthetised mice, aspirated virions infect lung epithelial cells [12]. However virions inhaled by non-anesthetised mice colonise only the upper respiratory tract before spreading to lymphoid tissue. An equivalent oral inoculum is non-infectious [13]. Therefore natural infection almost certainly proceeds via the upper respiratory tract. This requires the viral thymidine kinase [14] and ribonucleotide reductase [15], [16], implying that the primary target is a terminally differentiated cell.

*In vitro* MuHV-4 infection depends on virions binding to heparan sulfate (HS) [17], [18] via gp70 or gH/gL [19], [20]. Disrupting just one of these interactions, either genetically or with a blocking antibody, has relatively little effect on virion binding, but disrupting both causes a severe block [21]. The HS dependence of MuHV-4 also depends in a more complicated way on its gp150 [17]. Unlike gp70 and gH/gL, gp150 binds HS only weakly [19], and the main phenotype of gp150-deficient virions is not less binding to HS^+^ cells, but more binding to HS-deficient cells [17]. Gp150 disruption further rescues the infectivity of gL^−^gp70^−^ knockouts [21]. Thus gp150 acts as an HS-sensitive switch that normally inhibits non-HS ligand binding, for example by gB [22]; entry-associated antigenic changes in gp150 [23] may reflect its displacement to reveal non-HS binding; and when gp150 is missing, such binding is constitutively available. Analogous regulatory functions have been observed for the Bovine Herpesvirus-4 Bo10 [24] and the EBV gp350 [25].

Many herpesviruses bind to HS, and close functional correlates of the MuHV-4 gp70 and gH/gL exist in KSHV [26], [27]. Most epithelial cell lines display accessible HS. However *in vivo*, HS and the main epithelial proteoglycan, Syndecan-1, are basolateral [28], [29]. Limited apical HS expression correlates with reduced *in vitro* epithelial infection by cytomegalovirus [30]. An obvious question therefore is how HS binding herpesviruses efficiently enter new hosts when the apical epithelial surfaces they encounter lack HS. Here we used MuHV-4 as a tractable model with which to establish the *in vivo* relationship between host HS expression, virion binding, and infection.

## Results

### MuHV-4 infection localizes to the nasal septum and turbinates

**Figure 1:**
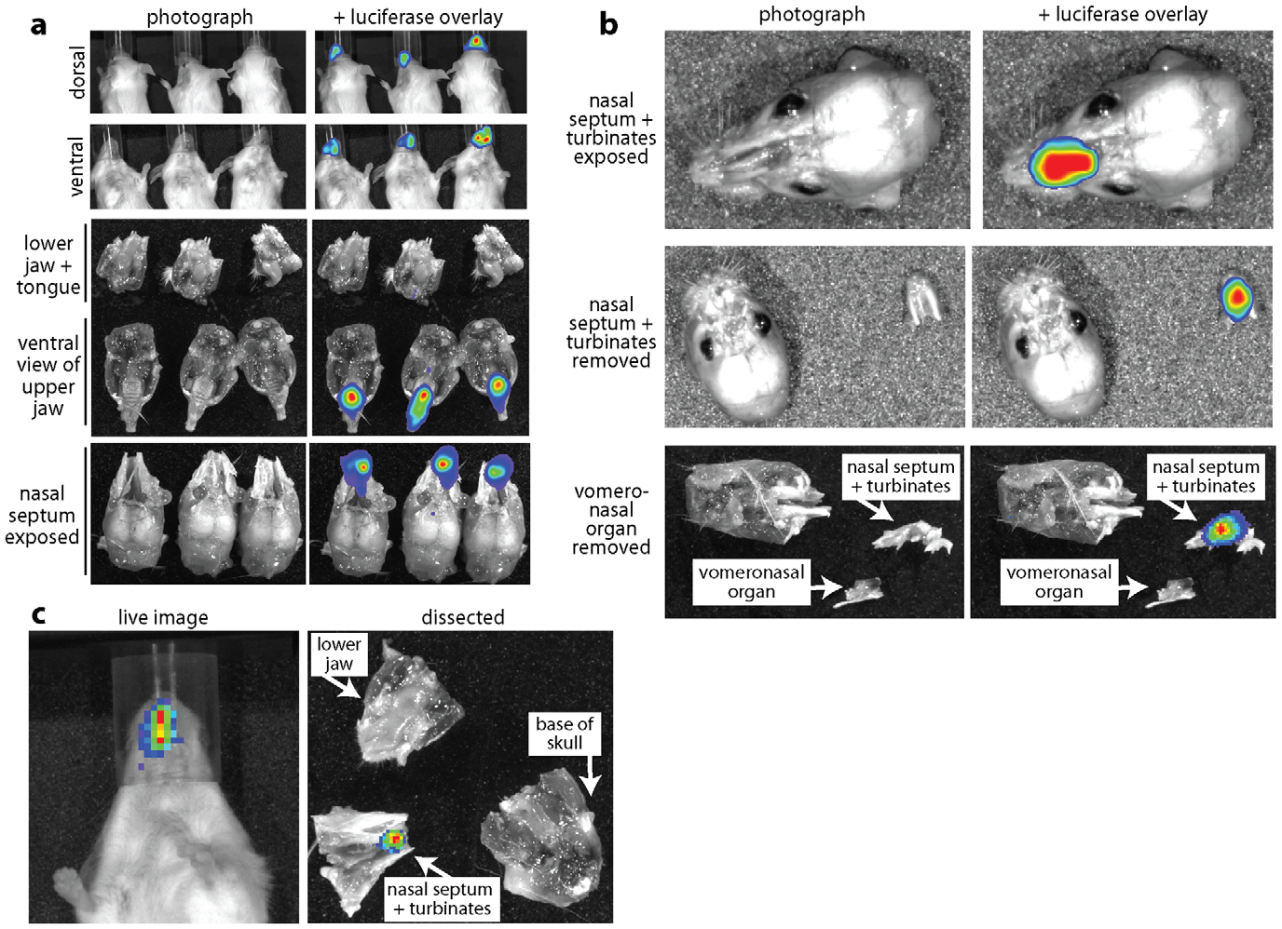
MuHV-4 infection localizes to the nasal septum and tubinates. **a.** BALB/c mice were allowed to inhale spontaneously a 5 µl droplet containing 10^4^ p.f.u. luciferase^+^ MuHV-4. 3 days later luciferase expression was identified by luciferin injection and CCD camera scanning of emitted light. The images shown are typical of >100 infections. Dissection localized the luciferase signal to a region occupied by the nasal septum/turbinates. **b.** BALB/c mice were infected and imaged as in **a**. Removing the septum/turbinates demonstrated that they accounted for all of the signal observed. Further dissection established that the vomeronasal organ lacked luciferase expression. Equivalent results were obtained in 10 mice. **c.** Luciferase^+^ MuHV-4 (10^6^ p.f.u. in 5 µl) was applied with a small brush to the external genitalia of female mice. Over the following 2 weeks, 8/12 co-caged male and female mice acquired nasal luciferase signals. Dissection again localized the luciferase signal to a region occupied by the nasal septum/turbinates. Representative images are shown.

Live imaging of virus-expressed luciferase has shown that MuHV-4 inhaled in a small volume by non-anesthetised mice infects the nose [13]. Post-mortem dissections (Fig. 1) localized this luciferase expression to the nasal septum and turbinates. Although much of an inhaled inoculum is likely to be swallowed, the tongue and oral mucosa remained luciferase-negative (Fig. 1a), consistent with oral virus being non-infectious [13]. The vomeronasal organ was also negative (Fig. 1b).

A 1 µl inoculum gave a similar picture to 5 µl (data not shown). The volume in which murid herpesviruses normally transmit is probably less still. To test this setting we applied luciferase^+^ MuHV-4 (10^6^ p.f.u.) to the external genitalia of female mice with a small brush, then co-caged these mice with naive males or females for 1 week. No mice showed genital infection, but 5/6 co-caged males and 3/6 co-caged females acquired nasal luciferase expression that again localized to the septum and turbinates. Fig. 1c shows an example. Therefore normal interactions between unrestrained mice allowed MuHV-4 to infect the upper respiratory tract.

### MuHV-4 selectively infects the olfactory neuroepithelium

**Figure 2:**
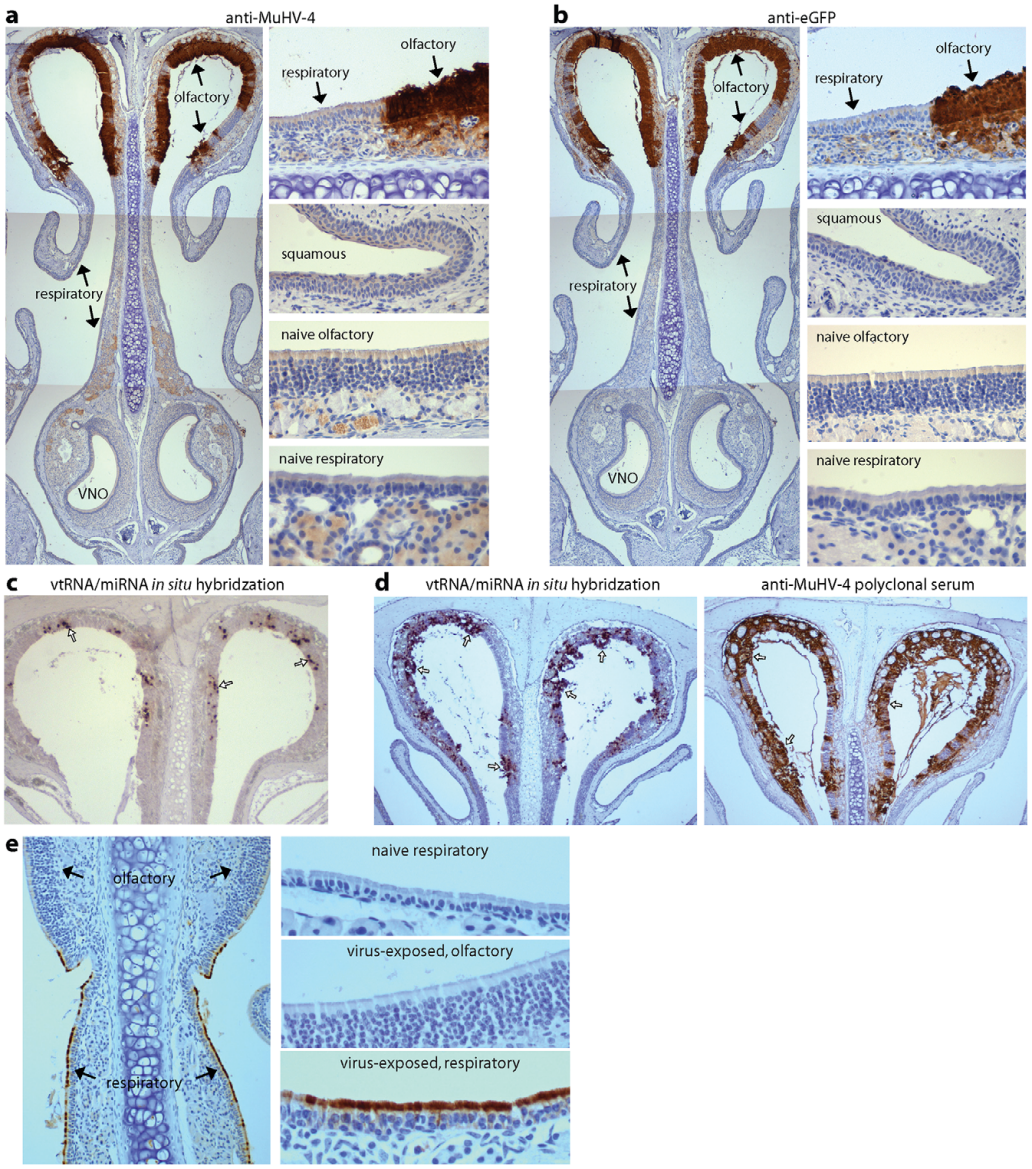
MuHV-4 infection localizes to the olfactory neuroepithelium. **a.** BALB/c mice were allowed to inhale a 5 µl droplet containing 10^6^ p.f.u. eGFP^+^ MuHV-4 and 3 days later analysed by immunostaining with a MuHV-4-specific polyclonal rabbit serum (brown) and counterstained with Mayer's Hemalum. The multi-layered main olfactory neuroepithelium and single-layered respiratory epithelium are indicated, as is the neuroepithelium of the vomeronasal organ (VNO). Equivalent staining was seen in >10 mice. Images enlarged ×5 in the right-hand panels show the sharp divide between virus^+^ neuroepithelium and virus^−^ respiratory epithelium; a representative area of (virus^−^) squamous epithelium; and background staining of sections from naive mice. **b** An adjacent section was stained for viral eGFP (brown), which is expressed independently of lytic cycle genes. Again the main olfactory neuroepithelium was virus^+^ and other sites virus^−^. **c.** BALB/c mice were infected with MuHV-4 (10^4^ p.f.u. in 5 µl) and 3 days later analysed for viral tRNA/miRNA expression by *in situ* hybridization of nose sections with a digoxigenin-labelled riboprobe. The arrows show examples of positive neuroepithelial staining. The respiratory epithelium remained negative. Similar results were obtained in 3 mice. **d.** BALB/c mice were infected with MuHV-4 (10^6^ p.f.u. in 5 µl) and 4 days later analysed either for viral tRNA/miRNA expression as in **c**, or for viral lytic antigen expression as in **a**. Arrows show examples of positive staining. **e.** BALB/c mice were allowed to inhale a 5 µl droplet containing 10^6^ p.f.u. influenza A/PR/8/34. 1 day later they were analysed by immunostaining with a polyclonal influenza-specific rabbit serum that recognizes predominantly the viral hemagglutinin. Only the respiratory epithelium was virus^+^. 5 more mice gave similar results.

The nose comprises squamous epithelium at the nostrils, then respiratory epithelium, and more rostrally neuroepithelium. We used immunostaining (Fig. 2) to identify more precisely the site of MuHV-4 infection. At day 3 post-inoculation (Fig. 2a) expression of the viral lytic antigens recognized by an immune rabbit serum [31] was essentially confined to the main olfactory neuroepithelium, with little or no staining of squamous or respiratory epithelia or the pheromone-sensing accessory olfactory neuroepithelium of the vomeronasal organ (which is vestigial in humans) [32].

To identify infection regardless of whether it was immediately productive, we stained sections for human cytomegalovirus IE1 promoter-driven viral eGFP, which is expressed independently of viral lytic antigens [33]. EGFP expression was also confined to the main neuroepithelium (Fig. 2b), as was the detection by *in situ* hybridization of viral tRNA/miRNAs, which are expressed in both lytic and latent infections [34] (Fig. 2c, 2d). Indeed *in situ* hybridization proved a less sensitive measure of infection than lytic antigen staining (Fig. 2d), arguing against there being sites of exclusively latent infection. Rather host entry was restricted to the main olfactory neuroepithelium.

Influenza virus delivered to the nose infected the respiratory epithelium and not the neuroepithelium (Fig. 2e). Therefore neuroepithelial infection was not just a consequence of virus inhalation, but was specific to MuHV-4.

### Both olfactory neurons and sustentacular cells are direct infection targets

**Figure 3:**
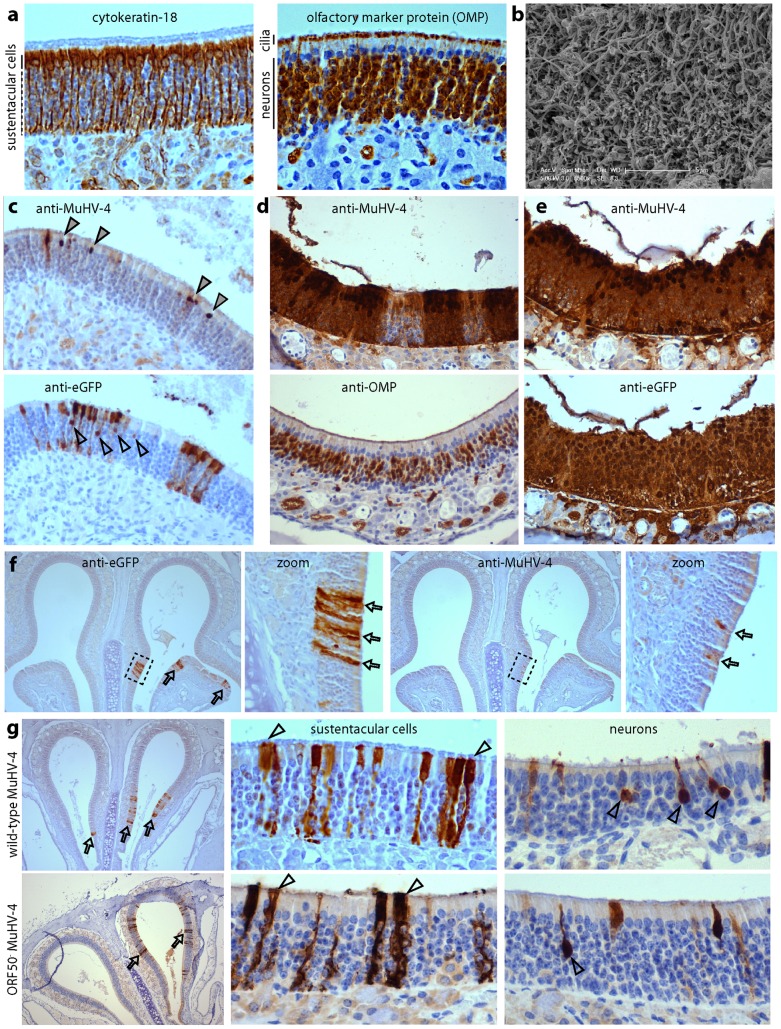
Both olfactory neurons and sustentacular cells are direct infection targets. **a.** The neuroepithelium of a naive BALB/c mouse was stained for cytokeratin-18 to reveal sustentacular cells (brown) and for olfactory marker protein to reveal neurons plus apical neuronal cilia (brown). Sections were counter-stained with Mayer's Hemalum. **b.** A scanning electron micrograph of the murine neuroepithelium showing the dense apical network of neuronal cilia. **c.** BALB/c mice were allowed to inhale eGFP^+^ MuHV-4 (10^6^ p.f.u.) and 1 day later analysed for viral lytic antigen (anti-MuHV-4) and eGFP expression with polyclonal sera (brown). The sections were counter-stained with Mayer's Hemalum. Filled arrowheads show examples of virus^+^ nuclei in the sustentacular cell layer; open arrowheads show examples of eGFP^+^ nuclei in the neuronal layer. **d.** At day 3 after infection as in **c**, the neuroepithelium was stained for MuHV-4 lytic antigens, or for OMP to show the distribution of neurons. Most virus^+^ nuclei were in the sustentacular cell layer between the neuronal nuclei and neuronal cilia. **e.** At day 3 after infection as in **c**, adjacent neuroepithelial sections were stained for MuHV-4 lytic antigens or virus-expressed eGFP. The former again showed positive nuclei mainly in the sustentacular cell layer, while the latter also stained nuclei throughout the neuronal layer. **f.** At day 1 after infection with eGFP^+^ MuHV-4 as in **c**, adjacent sections were stained for virus-expressed eGFP or lytic antigens. Each zoomed image shows the corresponding boxed region. The number of eGFP^+^ cells consistently exceeded by at least 3-fold the number of lytic antigen^+^ cells. The arrows show examples of positive staining. **g.** BALB/c mice were infected as in **c**, either with wild-type eGFP^+^ MuHV-4 or with an equivalent dose of ORF50^−^ (replication-deficient) eGFP^+^ MuHV-4. Samples taken 1 day later were stained for virus-expressed eGFP with a polyclonal serum. The arrows show examples of positive staining, which conformed mainly to the distribution of sustentacular cells. To the right, arrowheads show examples of sustentacular and neuronal staining at higher magnification.

The neuroepithelium comprises neurons, which mediate olfaction and express olfactory marker protein (OMP); sustentacular cells, which have a glial cell-like supporting role and express cytokeratin-18; and basal precursor cells (Fig. 3a, Fig.S1) [35]–[37]. The neuronal nuclei lie beneath those of the sustentacular cells, but neuronal dendrites project between the sustentacular cells, and from these long, fine, odorant binding cilia extend into the covering mucus. Thus the apical neuroepithelial surface consists largely of neuronal cilia (Fig. 3b).

One day after virus inhalation (Fig. 3c), the lytic antigen-specific rabbit serum stained almost exclusively sustentacular cells. Two days later lytic antigen expression was more extensive but retained the same predominantly sustentacular distribution, with few strongly MuHV-4^+^ nuclei in the OMP^+^ neuronal cell layer (Fig. 3d). The distribution of viral eGFP expression was less exclusive: at day 1 post-infection >80% of eGFP^+^ cells had a sustentacular distribution, but nuclei in the neuronal cell layer were also positive (Fig. 3c), and by day 3 (Fig. 3e) viral eGFP expression was abundant throughout the neuroepithelium. Thus both neurons and sustentacular cells were infected, but few neurons expressed viral lytic antigens. At day 1 post-inoculation, adjacent sections from individual mice contained 24.1±11.9 eGFP^+^ sustentacular cells and 6.5±5.7 lytic antigen^+^ sustentacular cells (mean ± SD, 6 mice) - Fig. 3f shows an example. Therefore even sustentacular infection was not uniformly lytic.

To determine the first cell type infected, we inoculated mice with MuHV-4 lacking its ORF50 (Rta) lytic transactivator. Such mutants express lytic genes only in complementing (ORF50^+^) cells [13], [38]. Lytic antigens were not visible by immunohistochemistry of ORF50^−^-infected mice (data not shown), but viral eGFP was expressed in both sustentacular cells and neurons (Fig. 3g). Therefore both were primary infection targets. At day 1 post-inoculation the sustentacular : neuron infection ratio was 5.5±3.9 (mean ± SD of 3 mice, counting 41–187 eGFP^+^ cells per mouse). A typical neuroepithelial cross-section contains 7.3±1.3 neurons per sustentacular cell (mean±SD, n = 50 cross-sections). Therefore despite neurons being more abundant, incoming virions predominantly infected sustentacular cells.

### Infant mice show a similar pattern of infection

**Figure 4:**
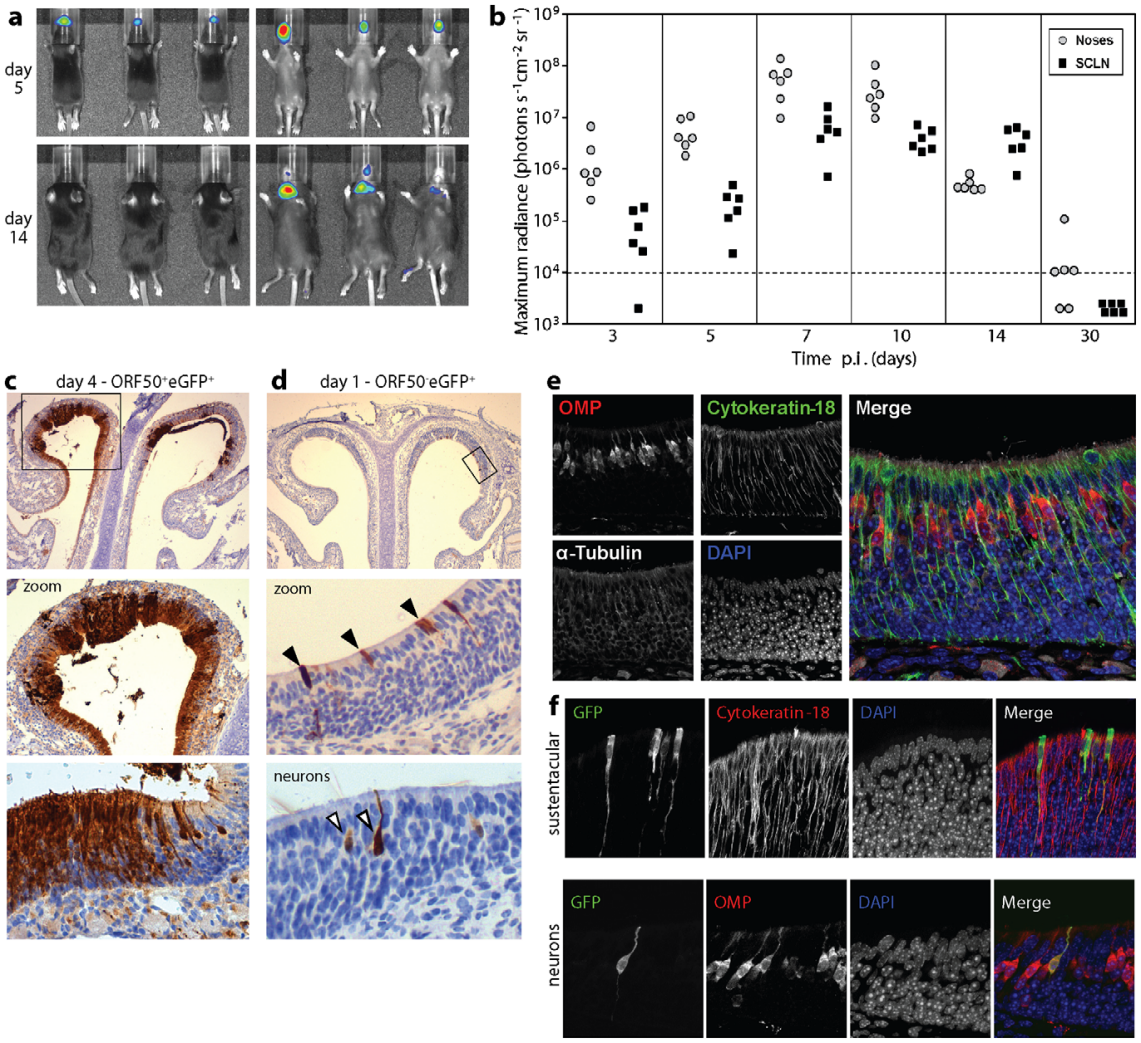
Direct neuronal and sustentacular infections also occur in infant mice. **a.** 1 week old C57BL/6 mice - BALB/c and C57BL/6 strains gave equivalent data in both adults and juveniles - were allowed to inhale luciferase^+^ MuHV-4 (10^4^ p.f.u. in 2 µl), and imaged for light emission 5 and 14 days later. The scale shows maximum radiance (photons sec^−1^ cm^−2^ steradian^−1^). The images are representative of >30 infections. **b.** Mice were infected as in a, then monitored serially by luciferin infection and CCD camera scanning of emitted light. Each point shows the result for 1 mouse. SCLN = superficial cervical lymph nodes (neck signal). The dashed line shows the lower limit of assay sensitivity. **c.** 1 week old C57BL/6 mice were allowed to inhale wild-type eGFP^+^ MuHV-4 (10^6^ p.f.u. in 2 µl) and 4 days later analysed by immunostaining for virus-expressed eGFP. The zoomed image shows the boxed region. The lower image shows a further magnified region of neuroepithelium with both sustentacular and neuronal eGFP expression. Equivalent results were obtained in 6 mice. **d.** 1 week old C57BL/6 mice were infected as in **c** but with replication-defective ORF50^−^eGFP^+^ MuHV-4 (10^6^ p.f.u., assayed on complementing cells). Infection was identified 1 day later by immunostaining for virus-expressed eGFP. The zoomed image shows the boxed region, with arrowheads indicating examples of positive (sustentacular cell) staining. The lower image shows a further magnified region of neuroepithelium with eGFP^+^ neurons. Equivalent results were obtained in 6 mice. **e.** Immunofluorescent staining of naive 1 week old C57BL/6 mice shows the characteristic distributions of OMP (neuronal axons, dendrites and cell bodies), α-tubulin (broadly expressed, including neuronal cilia) and cytokeratin-18 (sustentacular cells). Nuclei were counterstained with DAPI. The merged image shows distinct layers of neuronal cell bodies, sustentacular cells and neuronal cilia. **f.** 1 week old C57BL/6 mice were allowed to inhale ORF50^−^eGFP^+^ (10^6^ p.f.u. with complementation) and 1 day later analysed by immunofluorescent staining for eGFP, cytokeratin-18 and OMP. Nuclei were counterstained with DAPI. Co-localization appears yellow in the merged images. Equivalent results were obtained in 3 mice.

Herpesvirus infections often occur early in life. To establish whether MuHV-4 infects infant and adult mice in the same way, we allowed 1 week old mice to inhale a 2 µl luciferase^+^ virus droplet, then monitored infection by live imaging (Fig. 4a, 4b). As in adults [13], luciferase expression started in the upper respiratory tract and then spread to the draining (superficial cervical) lymph nodes. Immunostaining for virus-expressed eGFP at day 4 (Fig. 4c) showed widespread neuroepithelial infection with little or no respiratory epithelial infection, and replication-deficient MuHV-4 (Fig. 4d) again expressed eGFP in both sustentacular and neuronal cell distributions. Infant infections were therefore similar to adult.

We used immunofluorescence to identify primary infected cells more formally by co-staining. Fig. 4e shows OMP^+^ neurons and cytokeratin-18^+^ sustentacular cells in the neuroepithelium of an uninfected mouse. The apical neuronal cilia stained at best weakly for OMP, but were positive for α-tubulin, forming a distinct layer above the sustentacular cells. Fig. 4f shows neuroepithelial sections of infant mice infected 1 day earlier with replication-deficient (ORF50^−^) eGFP^+^ MuHV-4. Viral eGFP expression was seen in both OMP^+^ and cytokeratin-18^+^ cells. Thus incoming virions could infect both sustentacular cells and neurons. Counting 34–115 eGFP^+^ cells from each of 3 mice gave a sustentacular : neuron infection ratio of 3.6±3.4 (mean ± SD).

### Upper respiratory tract infection is HS-dependent

**Figure 5:**
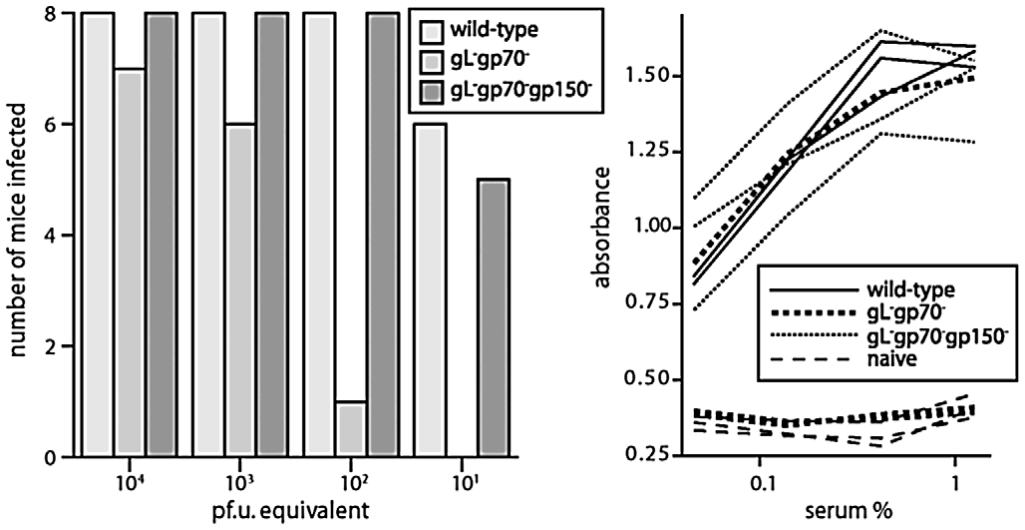
Neuroepithelial infection is HS-dependent. Adult C57BL/6 mice were allowed to inhale either wild-type, gL^−^gp70^−^ or gL^−^gp70^−^gp150^−^ virions in 5 µl. As gL^−^gp70^−^ MuHV-4 plaques poorly, the wild-type virus was titrated by plaque assay and all other virus stocks normalized to this by immunoblotting with MuHV-4-specific mAbs (Fig.S2). Groups of 8 mice were then exposed to virus and 1 month later scored as infected or not based on ELISA for virus-specific serum IgG. At 10^1^ and 10^2^ p.f.u. equivalents, gL^−^gp70^−^ MuHV-4 infected significantly fewer mice than either the wild-type or the HS-independent triple mutant (p<0.03 by 2-tailed Fisher's exact test). The graph on the right illustrates ELISA results for 3 mice per group from the 10^2^ p.f.u. equivalent dose, plus 3 naive controls. Note that the one mouse infected with gL^−^gp70^−^ virus shows an antibody response comparable to those infected with wild-type virus.

Selective olfactory neuroepithelial targeting by MuHV-4 implied that infection depended on one or more unique features of this site. *In vitro* MuHV-4 infection depends strongly on heparan sulfate (HS), and virions lacking HS binding proteins (gL^−^gp70^−^) poorly bind and infect transformed epithelial cells. They also poorly infect lungs [21]. To test the HS dependence of neuroepithelial infection we allowed unanesthetised mice to inhale different doses of wild-type or gL^−^gp70^−^ virions. The poor spread of gL^−^gp70^−^ MuHV-4 makes luciferase expression an insensitive read-out of infection. However it elicits a readily detectable antibody response [21]. We therefore assayed infection by ELISA for virus-specific serum IgG after 1 month (Fig. 5). The mutant virus (gL^−^gp70^−^) was approximately 100-fold less infectious than the wild-type by upper respiratory tract inoculation.

To address whether the gL^−^gp70^−^ infection defect might reflect functions of these proteins besides HS binding, we exploited the capacity of gp150 disruption to achieve functional reversion by making virion binding less HS-dependent [17]: gp150^+^ MuHV-4 follows a strict sequence of HS binding, then gp150 displacement, then HS-independent binding, but disrupting gp150 allows constitutive HS-independent binding, presumably by engaging a non-HS ligand that is normally inaccessible until gp150 is displaced. This restored the capacity of gL^−^gp70^−^ MuHV-4 to infect mice via the upper respiratory tract (Fig. 5).

Pre-incubating luciferase^+^ wild-type virions (2×10^5^ p.f.u./ml) with soluble heparin (1 mg/ml, 2 h, 23°C) before infecting adult mice (100 p.f.u. in 5 µl i.n.) also significantly reduced infection, as monitored by luciferase expression: virions incubated without heparin infected 7/8 mice; those incubated with heparin infected 1/8 mice (p<0.02 by 2-tailed Fisher's exact test). Thus neuroepithelial infection was HS-dependent.

### The olfactory neuroepithelium expresses apical HS

**Figure 6:**
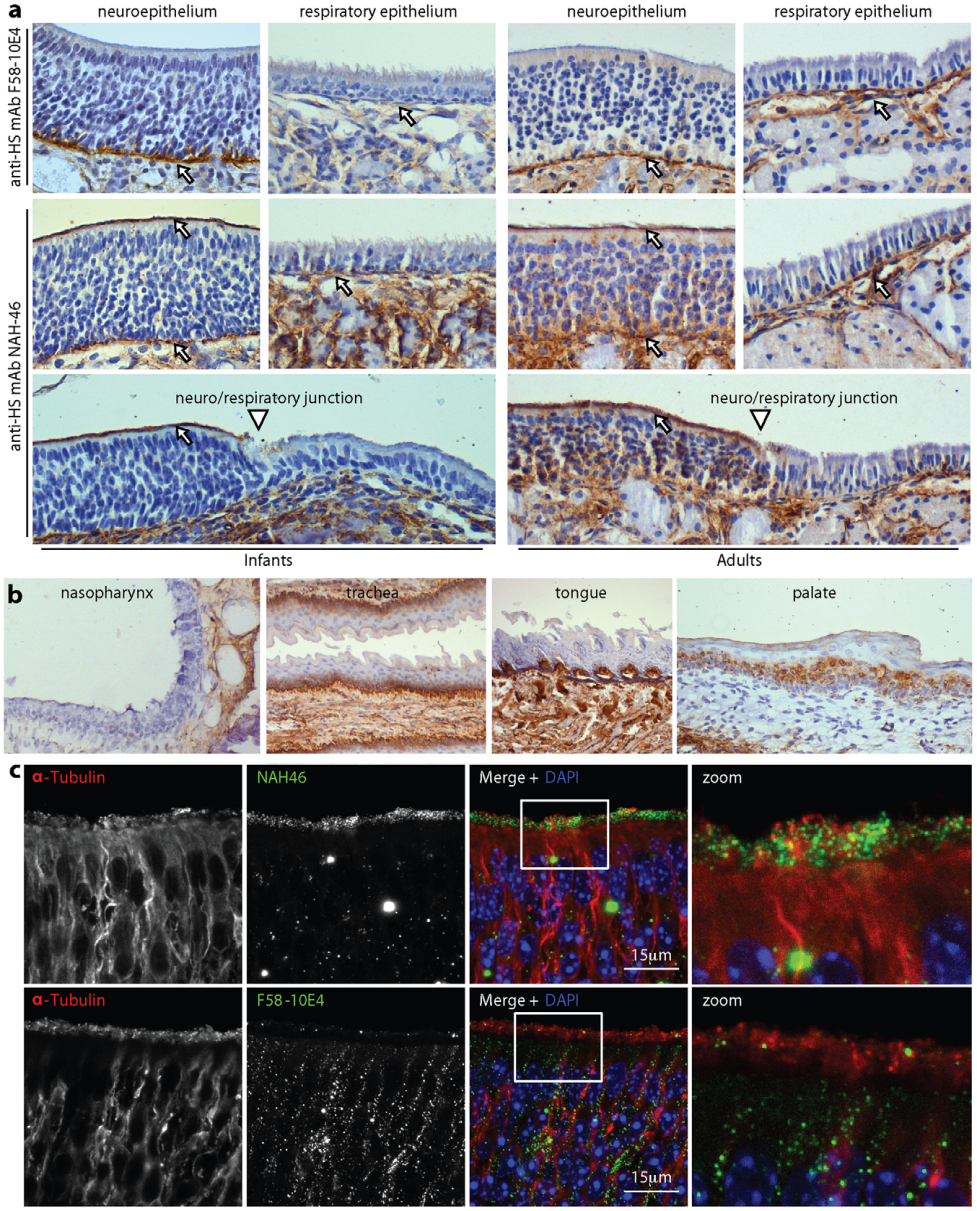
Identification of neuroepithelial HS. **a.** The neuroepithelia of infant and adult mice were analysed for HS expression by immunostaining with mAbs F58-10E4 (HS, sulfated) and NAH46 (HS, non-sulfated). Counter-staining was with Mayer's Hemalum. The arrows show areas of positive staining (brown). Arrowheads show the junction between neuroepithelium and respiratory epithelium. **b.** Tissues from naive adult mice were immunostained for HS using mAb NAH46 (brown) and counterstained with Mayer's hemalum (blue). **c.** Naive 1 week old mice were analysed for neuroepithelial HS expression by immunofluorescence with mAbs NAH46 and F58-10E4, and for α-tubulin to show the neuronal cilia. Nuclei were counter-stained with DAPI. The zoomed image showed the boxed region of the merge. NAH46 staining occupied the same region as the cilia, although their co-localization (yellow) was only partial.

We analysed neuroepithelial HS expression using 2 well-characterized mAbs (Fig. 6): F58-10E4 recognizes a sulfation-dependent HS epitope [39]; NAH46 recognizes a sulfation-independent epitope [40]. Most heparan shows partial sulfation [41]. However in both adult and infant mice, F58-10E4 stained only basolateral epithelial surfaces, while NAH46 also stained the apical neuroepithelium (Fig. 6a). A clear change from NAH46^−^ to NAH46^+^ was evident at the respiratory/neuroepithelial junction of both infant and adult mice (Fig. 6a). Other mucosal epithelia showed little or no apical staining by NAH46 (Fig. 6b) or by F58-10E4 (data not shown). Thus an apical expression of poorly sulfated heparan appeared to be unique to the olfactory neuroepithelium.

More detailed immunofluorescence imaging (Fig. 6c) showed some apical neuroepithelial F58-10E4 staining, but much less than with NAH46. The NAH46 staining occupied the same layer as the α-tubulin^+^ neuronal cilia. There was only limited co-localization between HS and α-tubulin itself, possibly because HS extends away from the cilial core or residual mucus limited antibody access. Nevertheless the apical HS was clearly associated with neuronal cilia rather than the underlying sustentacular cells.

**Figure 7:**
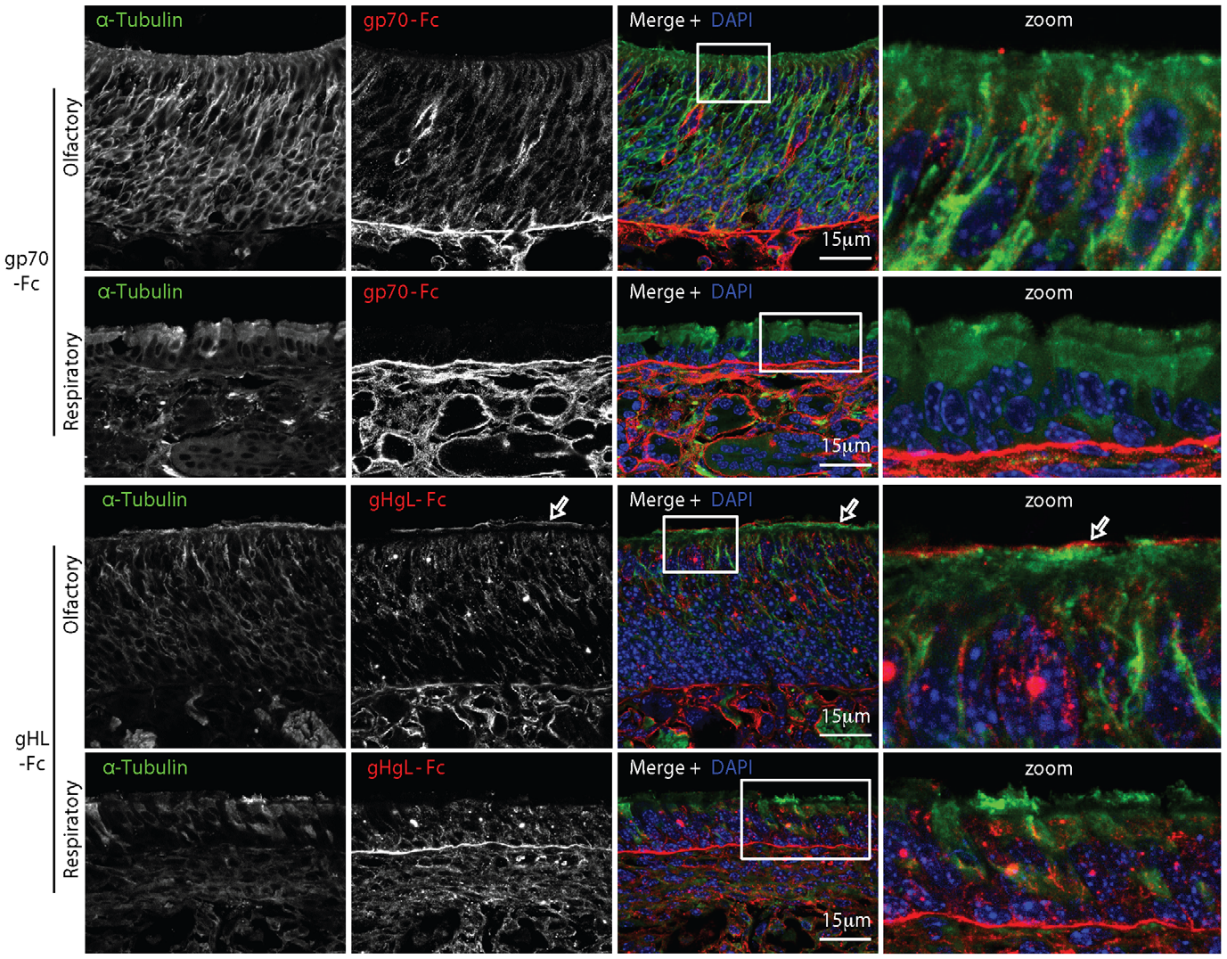
Neuroepithelial binding by virion glycoproteins. The virion HS binding proteins gp70 and gH/gL were expressed as IgG Fc fusions and used to stain neuroepithelial and respiratory epithelial sections from 1 week old naive mice. α-tubulin staining was used to identify the neuronal cilia. The zoomed image shows the boxed region of the merge. Colocalisation appears yellow. Similar results were obtained with 3 mice. The arrows show apical epithelial staining by gHgL-Fc.

We examined HS expression further by staining neuroepithelial sections with recombinant forms of the MuHV-4 gH/gL and gp70 extracellular domains fused to IgG Fc (Fig. 7). Both bind selectively to HS^+^ cells and are blocked from doing so by soluble heparin [20]. The equivalent glycoproteins of KSHV also show HS-dependent binding [26], [27]. Interestingly gp70-Fc and gHL-Fc showed distinct *in vivo* staining patterns: both bound to basolateral epithelia, but gHL-Fc, like mAb NAH46, bound also to the apical olfactory neuroepithelium, whereas gp70-Fc, like mAb F58-10E4, was restricted to basolateral epithelia. Thus gHL-Fc may bind better to poorly sulfated heparan. Many glycoproteins can carry HS. The best characterized are Syndecans 1–4 [41]. Syndecan-3 was abundant on neurons, and Syndecan-2 was expressed at sustentacular/neuronal junctions (Fig.S3). However no syndecans were expressed on the neuronal cilia.

### Virions bound to neuronal cilia reach both neurons and sustentacular cells

**Figure 8:**
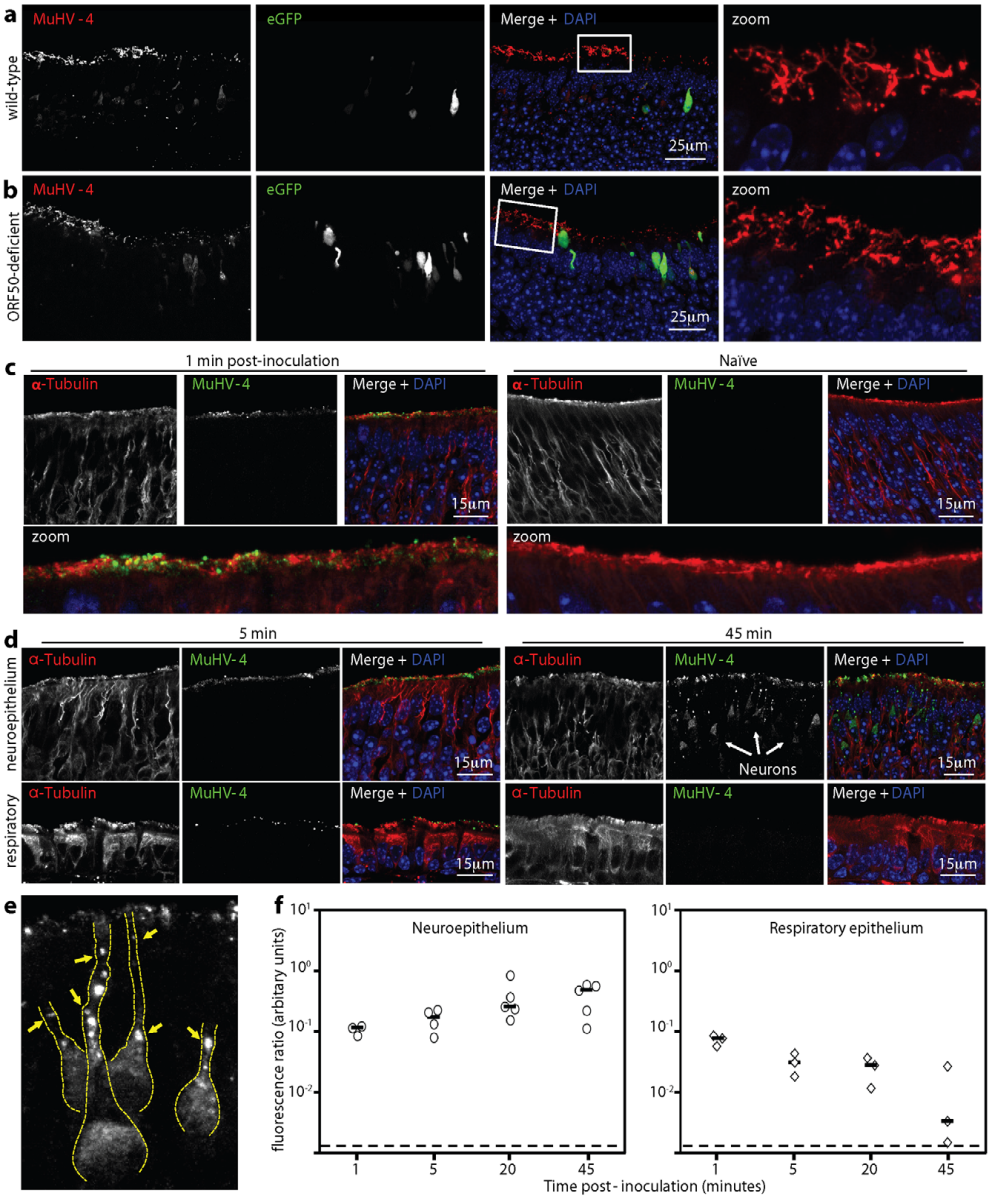
Virus binding to the olfactory neuroepithelium. **a.** 1 week old C57BL/6 mice were allowed to inhale wild-type eGFP^+^ MuHV-4 (10^6^ pf.u. in 2 µl). The next day neuroepithelial sections were analysed for viral lytic antigens and virus-expressed eGFP by immunofluorescent staining with polyclonal sera. Nuclei were counter-stained with DAPI. The zoomed image shows the boxed region of the merge. **b.** The same analysis was applied to ORF50^−^eGFP^+^ MuHV-4, which without complementation expresses eGFP but not new lytic antigens. **c.** 1 week old C57BL/6 mice were allowed to inhale MuHV-4 (10^6^ pf.u. in 2 µl) and 1 min later the same volume of PBS. They were then analysed by immunofluorescent staining of neuroepithelial sections for viral antigens. Neuronal cilia were visualised by staining for α-tubulin. Nuclei were counter-stained with DAPI. An equivalent section from a naive mouse is shown for comparison. **d.** Mice were infected as in **c** and examined for virus binding to the neuroepithelium and respiratory epithelium after 5 min or 45 min. The arrows at 45 min show viral antigen^+^ neurons. **e.** In a higher power image of the neuroepithelium 45 min after virus inhalation (10^6^ pf.u. in 2 µl), the dashed yellow lines outline neurons, as defined by α-tubulin staining, and the arrows show aggregated virion antigens along neuronal dendrites. **f.** After infection and staining as in **d**, virus binding was quantitated by counting MuHV-4^+^ pixels over a fixed area of apical epithelium and then normalizing by the α-tubulin signal of the same area. Each point shows the result for 3 sections from 1 mouse. The horizontal bars show medians. Comparison by Student's 2 tailed unpaired t test showed that neuroepithelial and respiratory epithelial binding were not significantly different at 1 min post-inoculation (p = 0.08) but at all subsequent time points neuroepithelial binding was significantly greater (p<0.05).

Neuroepithelial immunohistochemistry at 1 day after eGFP^+^ virus inoculation consistently showed at least 3-fold more eGFP^+^ cells than lytic antigen^+^ cells (Fig. 3). However confocal microscopy of equivalent immunofluorescent-stained neuroepithelia detected viral antigens more sensitively and revealed lytic antigens on neuronal cilia without associated eGFP expression (Fig. 8a). Infection with MuHV-4 that was unable to express new lytic antigens gave a similar picture (Fig. 8b). Thus the cilial staining came from input virions. It had a very different distribution to the productive infection of Fig. 3c–3e - for example cell nuclei were strongly stained in Fig. 3 and viral antigen-negative in Fig. 8.

Virus binding to the neuroepithelium was evident at just 1 min post-inoculation (Fig. 8c). A low level of respiratory epithelial binding was also evident at this time, possibly due to PBS inhalation incompletely rinsing off unbound virions, as this signal progressively declined with time, whereas neuroepithelial binding progressively increased. Fig. 8d shows that after 5 min neuroepithelial binding substantially exceeded respiratory epithelial binding. After 45 min the respiratory epithelium was viral antigen-negative, whereas the neuroepithelial virus had progressed into neurons. Detailed images (Fig. 8e) showed viral antigens within neuronal dendrites, consistent with endocytic transport [22]. Fig. 8f shows quantitatively how virus binding changed with time after inoculation.

**Figure 9:**
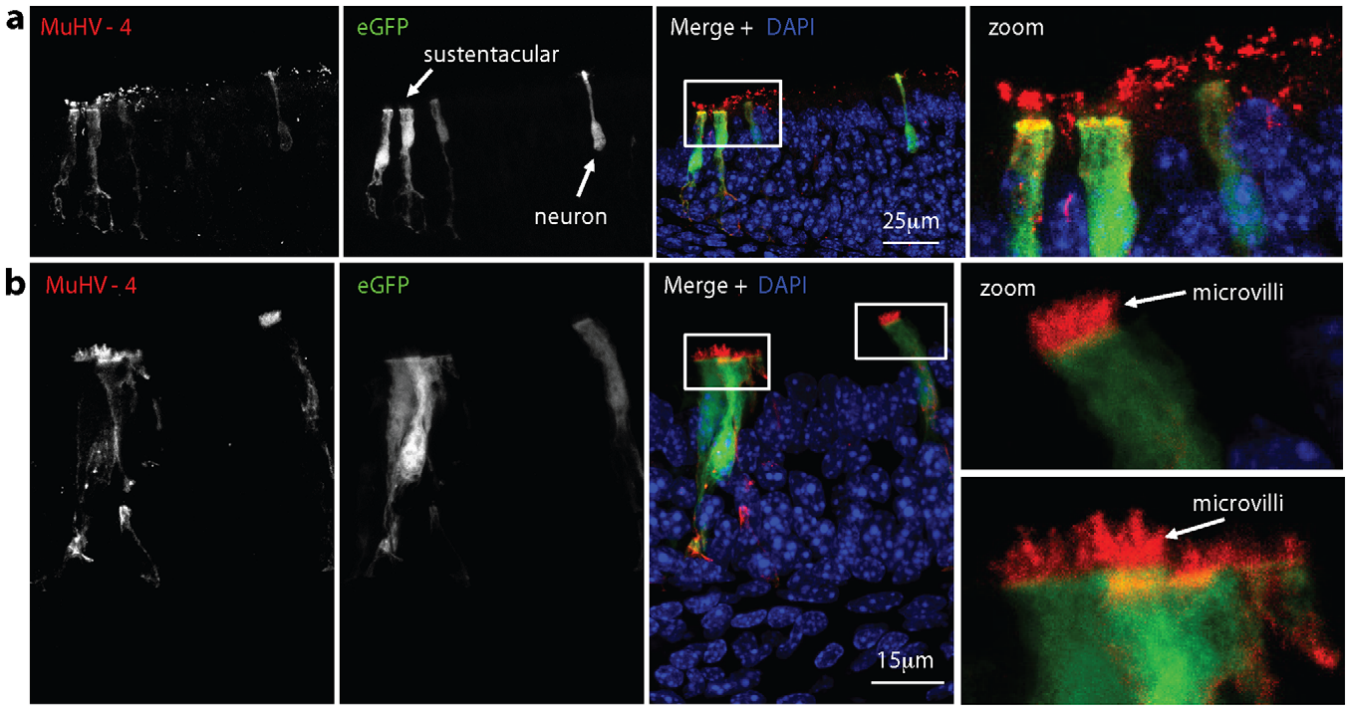
Virus uptake at the olfactory neuroepithelium. **a.** 1 week old C57BL/6 mice were allowed to inhale ORF50^−^eGFP^+^ MuHV-4 (10^6^ p.f.u. in 2 µl). The next day neuroepithelial sections were analysed for viral antigens and virus-expressed eGFP by immunofluorescent staining with polyclonal sera. Nuclei were counter-stained with DAPI. The zoomed image shows the boxed regions of the merge, with viral antigen^+^ neuronal cilia close to eGFP^+^ sustentacular cells. **b.** Mice were infected and analysed as in **a**. The zoomed images show eGFP^+^ sustentacular cells with viral antigen^+^ apical microvilli. The data are representative of >12 mice examined.

By 1 day post-inoculation replication-deficient MuHV-4 had infected many more sustentacular cells than neurons (Fig. 3), but after 45 min only neurons contained viral antigens. Sustentacular cells have relatively short apical microvilli that do not penetrate the olfactory mucus [42]. Thus limited sustentacular cell access to incoming virions appeared to mandate a more delayed infection. The distal dendrites onto which neuronal cilia converge form tight junctions with the apical surfaces of surrounding sustentacular cells [43]. One day after replication-deficient (ORF50^−^) MuHV-4 inhalation, viral antigen^+^ cilia were evident close to eGFP^+^ sustentacular cells (Fig. 9a), and many eGFP^+^ sustentacular cells had viral antigen^+^ microvilli (Fig. 9b). Thus after binding to the neuronal cilia, virions could reach sustentacular cell microvilli in an infection-competent form.

### Virions lacking HS binding show poor neuroepithelial uptake

**Figure 10:**
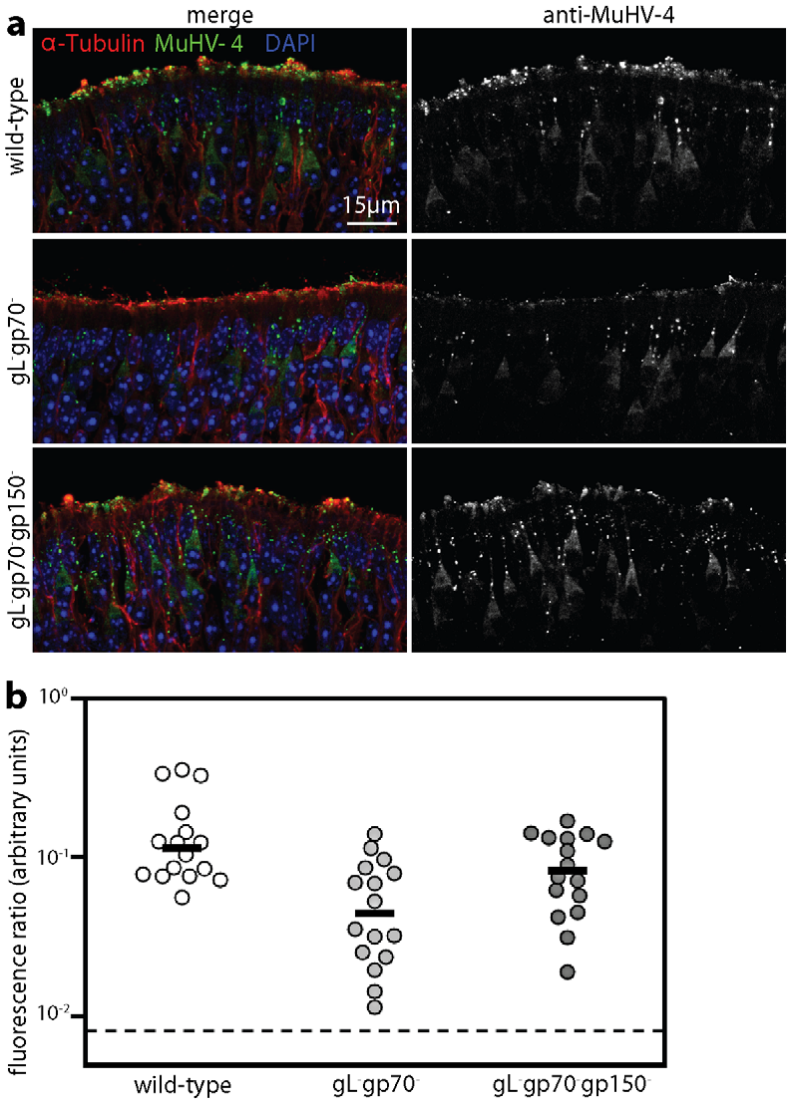
HS-dependent virion binding to the olfactory neuroepithelium. **a.** 1 week old mice were allowed to inhale wild-type, gL^−^gp70^−^, or gL^−^gp70^−^gp150^−^ virions (10^6^ p.f.u. equivalents in 2 µl). 45 min later the nasal passages were rinsed by PBS inhalation and neuroepithelial sections examined for viral antigens by immunofluorescent staining with a polyclonal immune serum. α-tubulin staining was used to visualise the neuroepithelial surface. Nuclei were counter-stained with DAPI. The images are representative of data from 3 mice per group. **b.** Viral antigen staining, as in **a**, was quantitated by counting MuHV-4 antigen^+^ pixels over representative fixed areas, and dividing by the α-tubulin^+^ pixel number. Each point shows the result for 1 section. Sections were pooled from 3 mice per group. The horizontal bars show medians. By Student's 2-tailed t test, gL^−^gp70^−^ virion binding was significantly reduced relative to wild-type (p<0.005) whereas gL^−^gp70^−^gp150^−^ virion binding was not (p = 0.3).

To relate neuroepithelial binding to infection (Fig. 5), we compared by viral antigen immunofluorescence infant mice 45 min after inhalation of wild-type, gL^−^gp70^−^ and gL^−^gp70^−^gp150^−^ virions (Fig. 10a). To visualize binding we used 1000 times more virus than for infection. Nevertheless fluorescence quantification (Fig. 10b) showed significantly less gL^−^gp70^−^ virion binding than wild-type, whereas gL^−^gp70^−^gp150^−^ and wild-type virions bound similarly. (The latter presumably bound to a down-stream, non-HS ligand.) The neuronal uptake of gL^−^gp70^−^ virions was difficult to quantitate accurately, but seemed proportionate to their binding. Thus HS engagement seemed to be important primarily for virion capture.

gL^−^gp70^−^gp150^−^ virions did not bind to the respiratory epithelium (data not shown), so the putative non-HS ligand engaged by them and normally covered by gp150 may also be restricted to the neuroepithelium. However gp150^+^ virions cannot engage such non-HS ligands without first engaging HS. HS availability therefore provided the proximate reason for olfactory neuroepithelial infection.

## Discussion

Herpesviruses enter new hosts at single sites, but can then disseminate and exit from multiple sites. Clinical presentation generally occurs some time after host entry, and corresponds more closely to the peak of host exit. Thus oropharyngeal symptoms and salivary virus shedding at presentation do not necessarily mean that host entry was also oral. Entry routes must be defined functionally. Most cell lines lack differentiation, polarization and a layer of mucus, while epithelial explants [44] involve tissue damage and can degenerate in unpredictable ways. The functional definition of host entry routes therefore requires *in vivo* infection models with realistic modes of virus uptake. Here we tracked host entry by MuHV-4 after its uptake by non-manipulated, unanesthetised mice.

Incoming virions infected the olfactory neuroepithelium. The apical cilia of olfactory neurons provided MuHV-4 with both a binding site and a path through the olfactory mucus. The small diameter of the cilia (100 nm) precludes an internal transport of 200 nm diameter virions, and probably even 100 nm diameter capsids. Virions may therefore instead travel externally along the cilia by retrograde transport [45], before endocytic infection [46] at the cilial pit, where they reach the terminal neuronal dendrite. Sustentacular infection by replication-deficient MuHV-4 implied that virions could also be captured intact from the cilia, consistent with the idea of external virion transport. Sustentacular uptake of virions in olfactory mucus [47] seemed less likely, as the mucus turns over only slowly and sustentacular infection was already abundant by 1 day post-inoculation. The neuronal cilia must receive a large inhaled antigen load, so removing particulate debris from them might be an important function of sustentacular cells that MuHV-4 exploits for host entry.

HS binding was a crucial first step in neuroepithelial infection: we found apical HS where infection occurred and not where it did not; this was confirmed by staining with recombinant gH/gL; virions lacking HS binding bound to and infected the neuroepithelium poorly; removing the need for virions to bind HS restored infection; and soluble heparin inhibited infection. Many herpesviruses bind to HS, and for many of these respiratory transmission is suspected [48]–[53]. Preliminary data show that Herpes simplex type 1, like MuHV-4, infects unanesthetised mice much better nasally than orally and targets the olfactory neuroepithelium (our unpublished data). Even EBV, which does not bind to HS, causes nasopharyngeal carcinoma, infects epithelial cells from the sphenoidal sinus [54] and infects rabbits after oral plus intranasal but not just oral inoculation [55]. MuHV-4 exploited neurons for host entry even though it is not classically neurotropic - dendritic cells transfer infection from the neuroepithelium to B cells [56]. Thus the olfactory neuroepithelium may provide many herpesviruses with an important entry portal.

HS binding is often considered to provide herpesviruses with merely non-specific capture prior to more critical protein binding. However it also provided a key component of MuHV-4 host entry. The exclusively basolateral HS expression of non-neuronal epithelia was consistent with their resisting infection by incoming virions. Their HS distribution would instead promote basolateral infection and apical virion release for efficient host exit. The normal function of olfactory HS may be to capture positively charged odorants or help support the neuronal cilia in the olfactory mucus. Its exploitation by MuHV-4 for host entry suggests that neuroepithelial-targeted, HS-based interventions could provide a general means of herpesvirus infection control.

Factors besides HS availability may also restrict MuHV-4 host entry to the olfactory neuroepithelium, as gp150 mutants did not infect more widely than wild-type despite being less HS-dependent. However such restrictions would operate only after HS binding, as gp150 normally prevents other viral ligand binding until HS itself has been engaged. Rather there may be multiple blocks to non-neuroepithelial infection, of which a need for HS is just the first. Whether down-stream events such as non-HS ligand engagement by gH/gL [57] or gB [22] can be also targeted to prevent neuroepithelial infection remains to be determined.

## Materials and Methods

### Ethics statement

All animal experiments were approved by the University of Cambridge ethical review board and by the UK Home Office under the 1986 Animal (Scientific Procedures) Act as Project Licence 80/2538.

### Mice

C57BL/6 and BALB/c mice (Harlan OLAC) were infected either as pups (1 week old) or as adults. Virus presented in 5 µl (adults) or 2 µl (pups) under light restraint without anesthesia was spontaneously inhaled. Standard infections used 10^4^ p.f.u. and those for histological analysis 10^6^ p.f.u. unless stated otherwise. For luciferase imaging, mice were injected intraperitoneally with luciferin (2 mg/mouse) then anesthetised with isoflurane and imaged for light emission with a CCD camera (Caliper Life Sciences). Luciferase images were analysed with Living Image software (Caliper Life Sciences).

### Cells and viruses

BHK-21 cells (American Type Culture Collection CCL-10), 293T cells (CRL-11268) NIH-3T3-ORF50 cells [13] and NIH-3T3-CRE cells [58] were propagated in Dulbecco's Modified Eagle's Medium, supplemented with 2 mM glutamine, 100 U/ml penicillin, 100 mg/ml streptomycin and 10% fetal calf serum (PAA laboratories). MuHV-4 was derived from a BAC-cloned viral genome [59]. ORF50^−^, luciferase^+^ [13], gL^−^gp70^−^ and gL^−^gp70^−^gp150^−^ viruses [21] have been described. The replication-deficient ORF50^−^ mutant was grown and titered by plaque assay on complementing NIH-3T3-ORF50 cells. Other viruses were grown and titered on BHK-21 cells [17]. Viruses were recovered from infected cell supernatants by ultracentrifugation (38,000× *g*, 90 min). Cell debris was removed by low speed centrifugation (500× *g*, 5 min). Any aggregates were then removed by filtration (0.45 µm). For viral eGFP expression, we used viruses that retained the HCMV IE1 promoter-driven eGFP of the BAC cassette [59]. The loxP-flanked BAC cassette was otherwise removed by virus passage through NIH-3T3-CRE cells. Influenza A/PR/8/34 was propagated in embryonated hen's eggs and titered by TCID_50_ assay on MDCK cells in serum-free medium with 1 µg/ml TPCK trypsin (Worthington) [60].

### Production of IgG Fc fusion proteins

The MuHV-4 gp70 HS binding site is contained within its first 2 short consensus repeat domains. To reconstitute this binding site in a soluble form we fused domains 1–3 to human IgG Fc [19]. The MuHV-4 gH/gL heterodimer is unstable without gB, but gH with gL fused to its C-terminus stably reproduces all known conformational epitopes of the virion gH/gL including its HS binding site. To test HS binding by gH/gL we fused this construct to human IgG Fc [20]. Fc fusion proteins were generated by transfecting expression plasmids into 293T cells with Fugene-6 and collecting supernatants 48 h and 72 h later. Fusion proteins were concentrated by binding to Protein A sepharose, released in glycine buffer pH 3, neutralized in Tris-HCl, dialysed against PBS, supplemented with 1% BSA and stored at −20°C.

### Immunohistochemistry

The whole nasal epithelium was removed post-mortem as a block of tissue bounded by the cartilaginous tip of the nose anteriorly, the orbits posteriorly, the zygomatic arches laterally, the palate ventrally and the nasal bones dorsally and fixed in PBS/4% formaldehyde (4°C, 24 h). Adult samples were decalcified by gentle agitation in 150 mM NaCl/50 mM TrisCl pH 7.2/270 mM EDTA for two weeks at 23°C, changing the solution every 3 days, then washed twice in PBS before dehydration in 70% ethanol and paraffin embedding. Infant samples were processed without decalcification. Paraffin-embedded samples were sectioned with a microtome (7 µm), then de-waxed in xylene and hydrated in graded ethanol solutions. Antigen retrieval was performed by microwaving 2×5 min in 10 mM NaCitrate pH 6/0.05% Tween-20. Endogenous peroxidase activity was quenched in PBS/3% H_2_O_2_ for 10 min. Sections were blocked with the Avidin/Biotin Blocking Kit (Vector Laboratories) and in PBS/2% BSA/2% normal serum from the species providing the secondary antibody (1 h, 23°C). MuHV-4 antigens were then detected with a rabbit immune serum [31], and eGFP with rabbit anti-eGFP pAb (Abcam). We detected OMP with a goat pAb (Santa Cruz Biotech), cytokeratin-18 with a rabbit pAb (Abcam), HSV-1 antigens with a rabbit pAb (Abcam), and influenza A/PR/8/34 with a rabbit pAb - kindly provided by Dr. P. Digard, University of Cambridge - that by immunoblotting recognizes predominantly the viral hemagglutinin. We detected HS with mAbs F58-10E4 and NAH46 (Seikagaku Corporation). After staining (18 h, 23°C), the sections were washed ×3 in PBS, incubated with biotinylated goat anti-rabbit IgG pAb, biotinylated goat anti-mouse pAb or biotinylated donkey anti-goat pAb (30 min, 23°C, all Vector Laboratories), washed ×3 in PBS, incubated with Vectastain Elite ABC Peroxidase system, washed ×3 in PBS, and developed with ImmPACT DAB substrate (Vector Laboratories). The sections were then counterstained with Mayer's Hemalum (Merck), dehydrated and mounted in DPX (BDH).

### *In situ* hybridization

Formaldehyde-fixed 7 µM tissue sections were de-waxed in xylene, rehydrated in graded ethanol solutions, then digested with proteinase K (100 µg/ml, 10 min, 37°C), and acetylated with 0.25% acetic anhydride in 0.1 M triethanolamine. Cells expressing viral tRNAs 1–4 were detected by hybridisation (18 h, 55°C) in 50% formamide, 1× SSC with a digoxigenin-labelled riboprobe transcribed from pEH1.4 [34]. The sections were washed in 0.1× SSC, 30% formamide at 58°C. Hybridized probe was detected with alkaline phosphatase-conjugated anti-digoxigenin Fab fragments plus BCIP/NBT substrate (Roche Diagnostics Ltd.).

### Immunofluorescence

Samples were fixed in 1% formaldehyde/10 mM sodium periodate/75 mM L-lysine (4°C, 24 h), equilibrated in 30% sucrose (4°C, 18 h), then frozen in OCT and sectioned (7 µm) on a cryostat. Sections were air dried (2 h, 23°C) and blocked with 2% serum/2% BSA/PBS (1 h, 23°C). For OMP and Cytokeratin-18 detection, antigen retrieval was performed by microwaving the sections (5 min) in 10 mM NaCitrate pH 6/0.05% Tween-20. Primary antibody incubations were as for immunohistochemistry. We detected α-tubulin with a rat mAb (Serotec). Staining with gp70-Fc and gHL-Fc was as for primary antibodies. After incubation sections were washed ×3 in PBS, then incubated (1 h, 23°C) with combinations of Alexafluor488- or 568-conjugated goat anti-mouse IgM pAb, Alexafluor568- or 633-conjugated goat anti-rat IgG pAb, Alexafluor488- or 568-conjugated goat anti-rabbit IgG pAb, Alexafluor568-conjugated goat anti-human IgG pAb, and Alexafluor568-conjugated donkey anti-goat IgG pAb (all from Invitrogen). After 3 further washes in PBS the sections were mounted in Prolong Gold + DAPI (Invitrogen). Fluorescence was visualised using a Leica TCS SP5 confocal microscope and analysed with ImageJ.

### Scanning electron microscopy

Tissues were fixed in 2% glutaraldehyde/0.1 M PIPES pH 7.4 (18 h, 4°C), rinsed in H_2_O, treated with 1% osmium ferricyanide (4°C, 18 h), rinsed in H_2_O, treated with 2% uranyl acetate/50 mM sodium maleate pH 5.5 (4°C, 18 h), rinsed in H_2_O and dehydrated in graded ethanol solutions. They were then dried (Polaron critical point dryer, Quorum/Emitech), coated with 10 nm gold (Quorum/Emitech K575X) and viewed in an FEI-Philips XL30 FEGSEM at 5 kv.

### ELISA

To measure MuHV-4-specific or HSV-1-specific serum IgG, virions were recovered from infected cell supernatants, then disrupted with 0.05% Triton X-100 and coated overnight at 4°C onto Maxisorp ELISA plates (Nalge Nunc). The plates were washed ×3 in PBS, blocked in PBS/1% BSA/0.1% Tween-20 (1 h, 23°C), incubated with 3-fold serum dilutions (1 h, room temperature), washed ×5 in PBS/0.1% Tween-20, incubated with alkaline phosphatase-conjugated goat anti-mouse IgG-Fc pAb (1 h, room temperature), washed ×5 in PBS/0.1% Tween-20, and developed with nitrophenylphosphate substrate (Sigma). Absorbance was read at 405 nm on a Biorad Benchmark Microplate Reader.

## Supporting Information

Figure S1 **Organization of the olfactory neuroepithelium.** Most neuroepithelial cells are bipolar neurons. Each has a terminal dendrite that terminates in a knob at the apical epithelial surface, from which emerge 10–15 long, fine, immotile cilia. These are embedded in the olfactory mucus. They carry the G protein-coupled odorant receptors responsible for olfaction. Each neuron also projects an axon to the olfactory bulb. The other major component of the neuroepithelium is sustentacular cells. These too span the epithelium, and their nuclei form a layer above those of the neurons. Sustentacular cell functions are poorly defined, but a glial cell-like supporting role for the neurons is likely. The presence of apical microvilli suggests an absorptive function, perhaps related to detoxification and odorant removal. Basal progenitor cells give rise to both olfactory neurons and sustentacular cells, and allow slow regeneration of the neuroepithelium after damage.
(TIF)

Figure S2 **Normalisation of virus stocks by protein content.** The protein content per p.f.u. of filtered wild-type (WT), gL**^−^**gp70^−^, and gL^−^gp70^−^gp150^−^ MuHV-4 stocks was determined by immunoblot against thymidine kinase (TK; mAb MG-4A5), gN (mAb 3F7), the C-terminal half of gB (mAb MG-4D11) and the capsid protein products of ORF17 (mAb 150-7D1). The signal for full-length gB (gB-FL) is weak because most virion gB is cleaved. gL^−^gp70^−^ stocks contained approximately 20 times more protein per p.f.u. than WT, because this virus binds poorly to cells and so plaques poorly. The protein/p.f.u. ratio of gL^−^gp70^−^gp150^−^ MuHV-4 was equivalent to WT because gp150 disruption rescues the infectivity of gL^−^gp70^−^ virions.
(TIF)

Figure S3 **Syndecan staining of the olfactory neuroepithelium.** Neuroepithelial sections were stained with mAbs to each syndecan (brown) and counter-stained with Mayer's hemalum (blue). Syndecan-1 was exclusively basolateral, consistent with published studies. Syndecan-2 localized to tight junctions between the neurons and sustentacular cells. Syndecan-3 (neuro-syndecan) outlined the neuronal cell bodies and dendrites. Syndecan-4 was seen on sustentacular cells. However none appeared to be expressed on the apical neuronal cilia.
(TIF)
