# Supplementary figures and images for: A Heparan-Dependent Herpesvirus Targets the Olfactory Neuroepithelium for Host Entry
Source: PLoS Pathog. 2012 Nov 1;8(11):e1002986. doi: 10.1371/journal.ppat.1002986 (PMC3486907; doi:10.1371/journal.ppat.1002986)

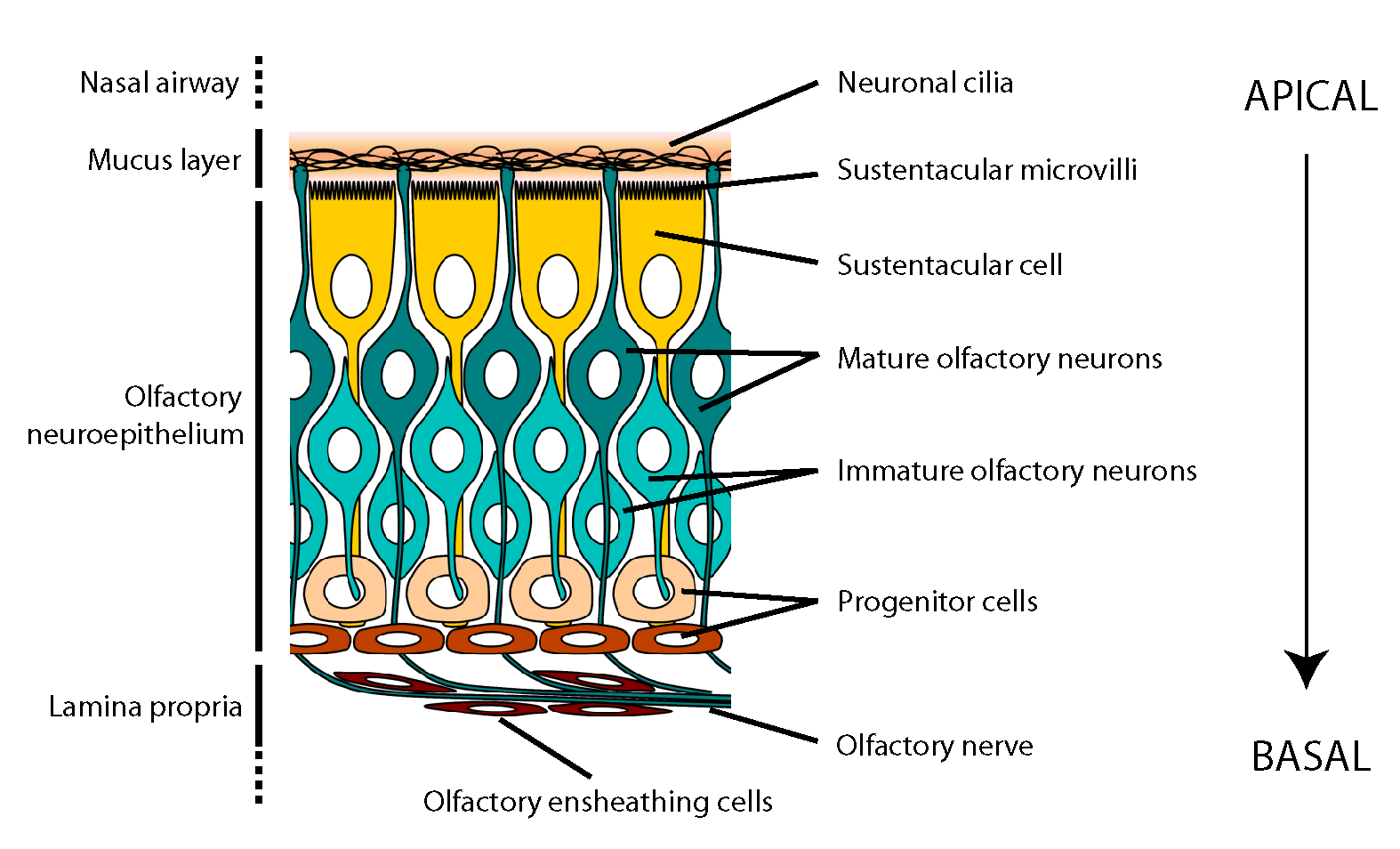

Supplement: Figure S1 — Organization of the olfactory neuroepithelium. Most neuroepithelial cells are bipolar neurons. Each has a terminal dendrite that terminates in a knob at the apical epithelial surface, from which emerge 10–15 long, fine, immotile cilia. These are embedded in the olfactory mucus. They carry the G protein-coupled odorant receptors responsible for olfaction. Each neuron also projects an axon to the olfactory bulb. The other major component of the neuroepithelium is sustentacular cells. These too span the epithelium, and their nuclei form a layer above those of the neurons. Sustentacular cell functions are poorly defined, but a glial cell-like supporting role for the neurons is likely. The presence of apical microvilli suggests an absorptive function, perhaps related to detoxification and odorant removal. Basal progenitor cells give rise to both olfactory neurons and sustentacular cells, and allow slow regeneration of the neuroepithelium after damage. (TIF) [file ppat.1002986.s001.tif]

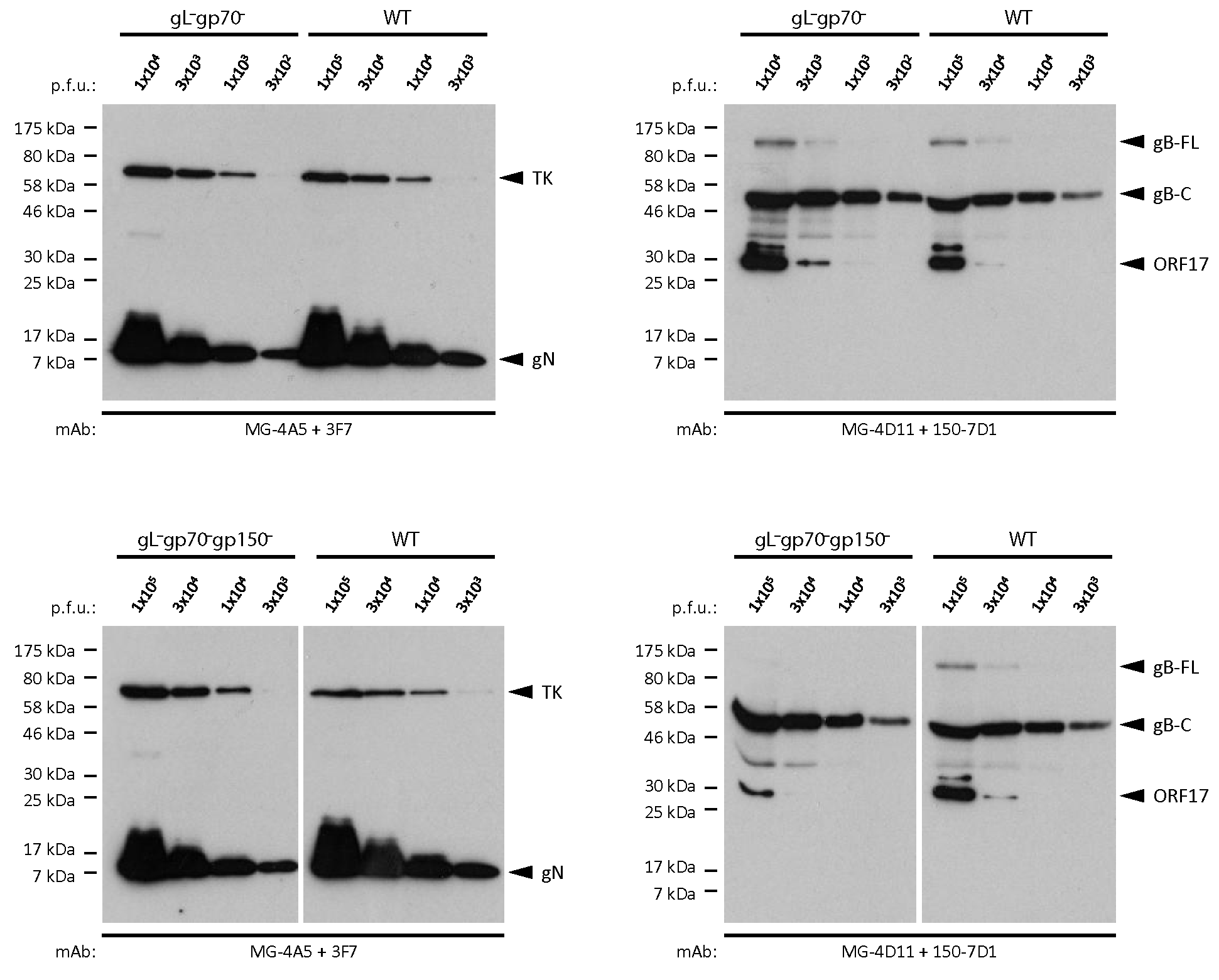

Supplement: Figure S2 — Normalisation of virus stocks by protein content. The protein content per p.f.u. of filtered wild-type (WT), gL−gp70−, and gL−gp70−gp150− MuHV-4 stocks was determined by immunoblot against thymidine kinase (TK; mAb MG-4A5), gN (mAb 3F7), the C-terminal half of gB (mAb MG-4D11) and the capsid protein products of ORF17 (mAb 150-7D1). The signal for full-length gB (gB-FL) is weak because most virion gB is cleaved. gL−gp70− stocks contained approximately 20 times more protein per p.f.u. than WT, because this virus binds poorly to cells and so plaques poorly. The protein/p.f.u. ratio of gL−gp70−gp150− MuHV-4 was equivalent to WT because gp150 disruption rescues the infectivity of gL−gp70− virions. (TIF) [file ppat.1002986.s002.tif]

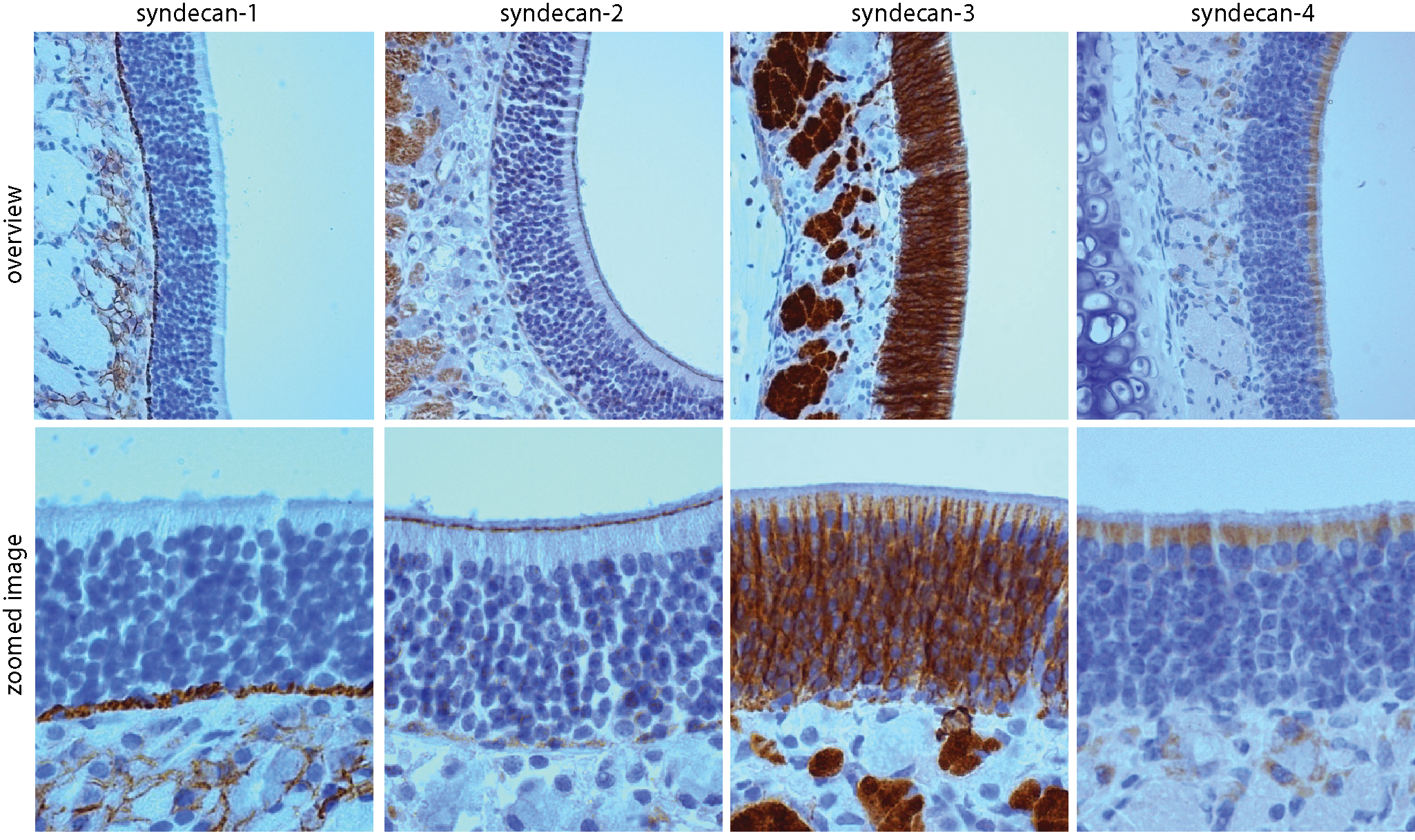

Supplement: Figure S3 — Syndecan staining of the olfactory neuroepithelium. Neuroepithelial sections were stained with mAbs to each syndecan (brown) and counter-stained with Mayer's hemalum (blue). Syndecan-1 was exclusively basolateral, consistent with published studies. Syndecan-2 localized to tight junctions between the neurons and sustentacular cells. Syndecan-3 (neuro-syndecan) outlined the neuronal cell bodies and dendrites. Syndecan-4 was seen on sustentacular cells. However none appeared to be expressed on the apical neuronal cilia. (TIF) [file ppat.1002986.s003.tif]
